# Deltoid Branch of Thoracoacromial Vein: A Safe Alternative Entry Vessel for Intravenous Port Implantation

**DOI:** 10.1097/MD.0000000000000728

**Published:** 2015-05-01

**Authors:** Ta-Wei Su, Ching-Feng Wu, Jui-Ying Fu, Po-Jen Ko, Sheng-Yueh Yu, Tsung-Chi Kao, Hong-Chang Hsieh, Ching-Yang Wu

**Affiliations:** From the Division of Thoracic and Cardiovascular Surgery (TWS, CFW, PJK, SYY, TCK, HCH, CYW), Department of Surgery, Chang Gung Memorial Hospital, Chang Gung University; and Division of Chest and Critical care (JYF), Department of Internal Medicine, Chang Gung Memorial Hospital, Chang Gung University, Kweishan, Taoyuan, Taiwan.

## Abstract

An entry vessel is crucial for intravenous port implantation. A safe alternative entry vessel that can be easily explored is crucial for patients without feasible cephalic vein or for those who need port reimplantation because of disease relapse. In this study, we tried to analyze the safety and feasibility of catheter implantation via the deltoid branch of the thoracoacromial vein.

From March 2012 to November 2013, 802 consecutive oncology patients who had received intravenous port implantation via the superior vena cava were enrolled in this study. The functional results and complications of different entry vessels were compared.

The majority of patients (93.6%) could be identified as thoracoacromial vessel. The deltoid branch of the thoracoacromial vein is located on the medial aspect of the deltopectoral groove beneath the pectoralis major muscle (85.8%) and in the deep part of the deltopectoral groove (14.2%). Due to the various calibers employed and tortuous routes followed, we utilized 3 different methods for catheter implantation, including vessel cutdown (47.4%), wire assisted (17.9%), and modified puncture method (34.6%). The functional results and complication rate were similar to other entry vessels.

The deltoid branch of the thoracoacromial vein is located in the neighborhood of the cephalic vein. The functional results of intravenous port implantation via the deltoid branch of the thoracoacromial vein are similar to other entry vessels. It is a safe alternative entry vessel for intravenous port implantation.

## BACKGROUND

An entry vessel is crucial for intravenous port implantation. The ideal entry vessel has consistent location and adequate caliber. Three different entry vessels candidates, including cephalic vein, subclavian vein, and internal jugular vein, can be utilized for superior vena cava catheter implantation. The cephalic vein is the most common and easiest choice because of its relatively consistent location and large caliber. However, in about 18% of patients the cephalic vein cannot be identified and another entry vessel is needed for catheter implantation.^1^

Other common alternative entry vessels for catheter implantation are the subclavian and internal jugular veins. In the case of the subclavian vein, the risk of hemopneumothorax and pinch-off symptoms cannot be totally eliminated even for an experienced surgeon.^2–5^ In the case of the internal jugular vein, the risk of vessel injury still remains, even under echo-guidance assist and vessel repair is warranted.^6^ Furthermore, an additional subcutaneous tunnel between the entry site and the injection chamber is necessary in order to embed the catheter. Postoperation pain can last from days to weeks, decreasing the patient's quality of life.^7^ Therefore, the above mentioned alternative entry vessels are not ideal for catheter implantation. However, a safe alternative entry vessel that can be easily explored is crucial for patients without feasible cephalic vein or for those who need port reimplantation because of disease relapse. The deltoid branch of the thoracoacromial artery and vein are located in the neighborhood of the deltopectoral groove and have been reported as recipient vessels of reconstructed muscle or free jejunum flap.^8–12^ From an anatomic view, a cadaveric study done by Loukas et al revealed that 65.2% of the cephalic vein travels with the deltoid branches of the thoracoacromial trunk.^8^ These characteristics suggest the deltoid branch of the thoracoacromial vein as an entry vessel candidate for catheter implantation. In this study, we try to identify the availability and feasibility of catheter implantation in the deltoid branch of the thoracoacromial vein.

## MATERIALS AND METHODS

### Patients

From March 2012 to November 2013, 809 consecutive oncology patients received intravenous port implantation. Seven patients who received intravenous port implantation via the inferior vena cava route were excluded. We collected all data from medical records and follow up from these patients until January 2014. All detailed information was documented in the operative permit and explained by preoperative verbal explanation. All data were deidentified prior to analysis. This study has been approved by the Ethics Committee of Chang Gung Memorial Hospital, under the institutional review number 100–4193A3.

### Medical Decision

The decision-making process regarding the entry vessel is as follows.^4^ All patients underwent exploration for cephalic vein and deltoid branch of thoracoacromial vein from the same incision. The first preference for catheter implantation was the cephalic vein; however, if it was absent or fibrotic and could not be utilized for implantation, the second choice was the deltoid branch of the thoracoacromial vein. The internal jugular vein was considered the last choice for entry vessel only if the cephalic vein and the deltoid branch of the thoracoacromial vein were both absent. The subclavian vein was not utilized for catheter implantation because of the risk of iatrogenic pneumohemothorax and pinch-off syndrome.

### Operative Method

**FIGURE 1:**
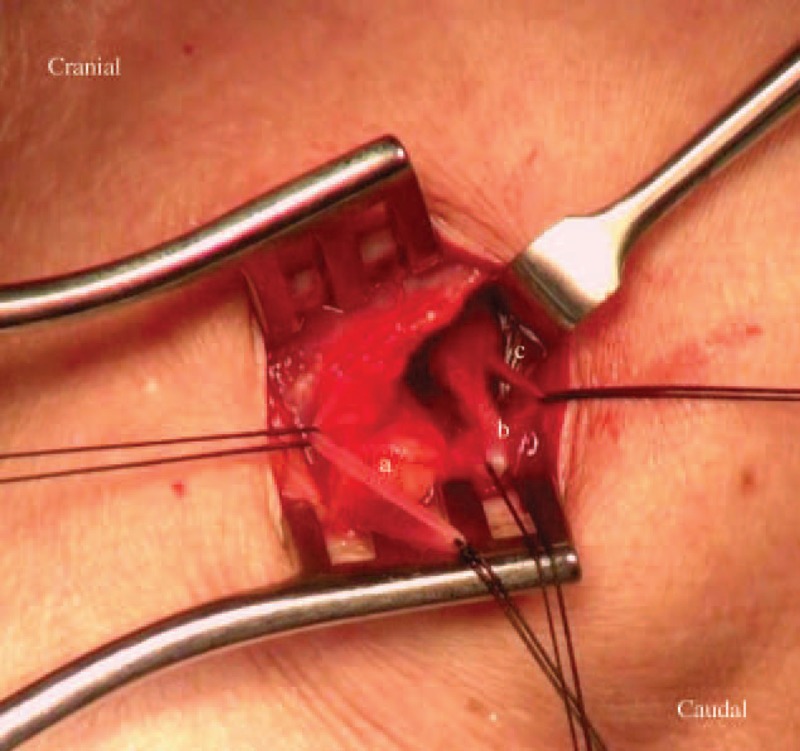
(A) Cephalic vein. (B) Thoracoacromial artery, deltoid branch. (C) Thoracoacromial vein, deltoid branch.

**FIGURE 2:**
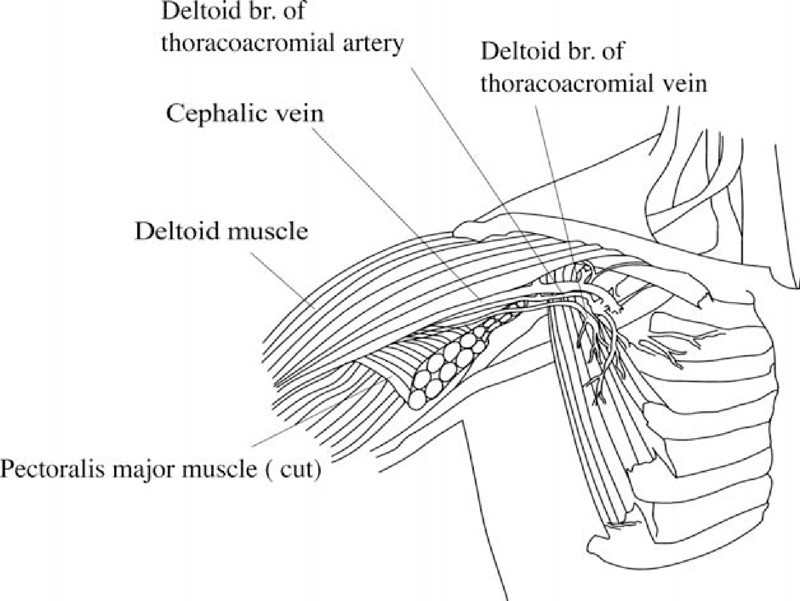
Schematic of the position of cephalic and deltoid branch of thoracoacromial vein.

**FIGURE 3:**
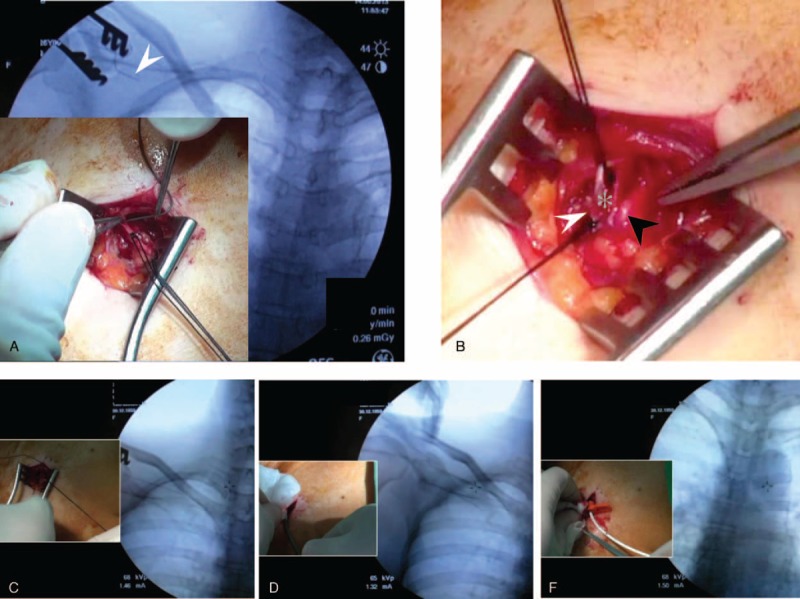
(A) Cephalic vein fibrosis such that metallic wire could not pass the lesions (white arrow). (B) Left: Ligation of the fibrotic cephalic vein (white arrow); right: exploration of deltoid branch of thoracoacromial artery (black arrow) and vein (gray star). (C) Utilization of metallic wire to cannulate the vessel and establish an entry route for the catheter. (D) Utilization of peel-apart dilator sheath over the wire in order to create a subcutaneous tunnel for catheter implantation. (E) Implantation of the catheter via sheath to an adequate tip location.

We performed local anesthesia in the subclavicular area and created a 2-cm incision for vessel exploration. We explored the cephalic vein and the deltoid branch of the thoracoacromial vein for every patient via the same wound (Figures 1 and 2). The cephalic vein and deltoid branch of the thoracoacromial vein were explored first. Vessel cutdown or endovascular wire–assisted technique was utilized according to vessel distribution and caliber. The vessel cutdown method, that is, distal ligation of the vessel and proximal stay suture to secure the venotomy site, was utilized for vessels with caliber larger than the catheter. Endovascular wire–assisted technique was used for catheter implantation if the vessel was of small caliber, had sharp angles, or involved tortuous routes during catheter implantation. Two different metallic wires, V-18 Control Wire (0.018 , 200 cm, Boston Scientific, Natick, MA) and Guide Wire M (0.035 in, 150 cm Terumo Cooperation, Tokyo, Japan), were chosen, according to the vessel caliber. As long as the metallic wire could establish a route for catheter implantation, we were able to slide the catheter over the wire or a subcutaneous dilator with a peel-able sheath could be used for a subcutaneous tunnel creation along the native vessel route (Figure 3). The catheter could be implanted via a peel-apart sheath under fluoroscopy. Finally, we created a subcutaneous pocket between the subcutaneous fat and fascia of the pectoralis major for placement of the injection chamber.

### Statistics

All the collected clinicopathologic factors were first analyzed with univariate analysis. Categorical variables were compared using χ^2^ or Fisher exact tests. A p-value less than 0.05 was considered statistically significant. Reported confidence intervals (CI) are assumed to have a coverage probability of 95%. All the analyses were performed using SAS, version 9 (SAS Institute, NC).

## RESULTS

From March 2012 to November 2013, 802 consecutive oncology patients received intravenous port implantation via the superior vena cava route. The descriptive characteristics of the patients are listed in Table 1. We explored the deltopectoral groove and nearby area in order to identify the location and pattern of the thoracoacromial vessel in all patients. Ninety-five patients were unable to tolerate the procedure because of relatively poor general condition such as dyspnea or coagulopathy. Seven hundred seven patients received vessel explorations, with only 45 patients (6.4%) in whom the deltoid branch of the thoracoacromial vein could not be identified during exploration on account of abundant adipose tissue or intolerable pain. The remaining 644 patients (91%) were identified to have a pattern of a thoracoacromial vessel with 1 artery and 1 vein. Furthermore, the locations of the deltoid branch of the thoracoacromial vein were the medial aspect of the deltopectoral groove, beneath the pectoralis major muscle (85.8%) and in the deep part of the deltopectoral groove (14.2%).

**TABLE 1:**
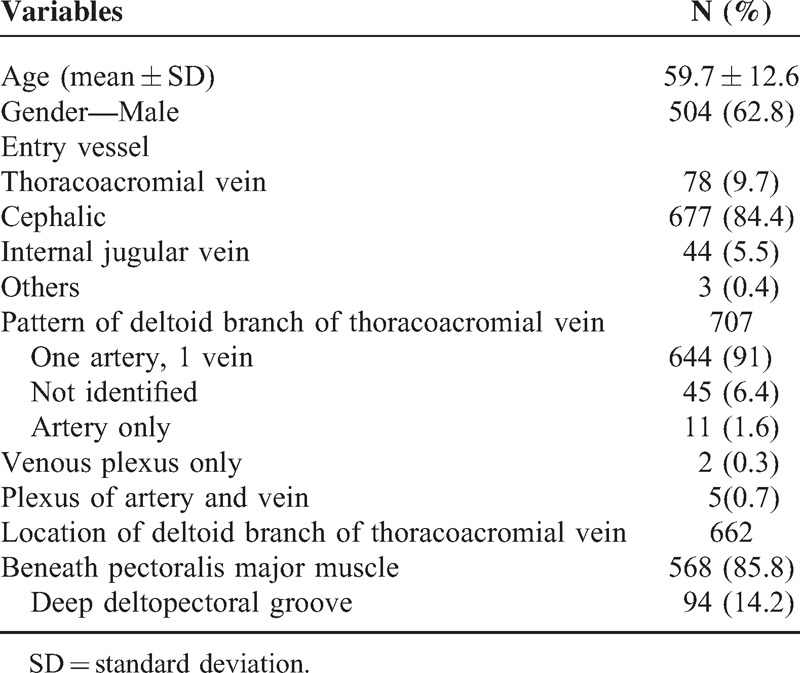
Descriptive Statistics

In total, 78 patients received intravenous port implantation via the deltoid branch of the thoracoacromial vein (Table 2). The mean operation time was 44.1 minutes. Thirty-seven patients (37/78, 47.4%) received direct catheter implantation via the thoracoacromial vein using the vessel cutdown method. Fourteen patients (17.9%) were found to have a tortuous vessel route and required metallic wire in order to establish an entry route and permit catheter implantation over the wire. Twenty-seven patients (34.6%) were found to have small vessel caliber with tortuous vessel route. For these patients, we utilized a small caliber metallic wire to establish a route and subcutaneous dilator with a peel-able sheath that had been used for subcutaneous tunnel creation along a native vessel route. After the dilator was removed, the catheter could be implanted into the vessel via a peel-able sheath. The functional results and complication rate of the thoracoacromial vein were similar to implantations via the cephalic and internal jugular vein (Table 3).

**TABLE 2:**
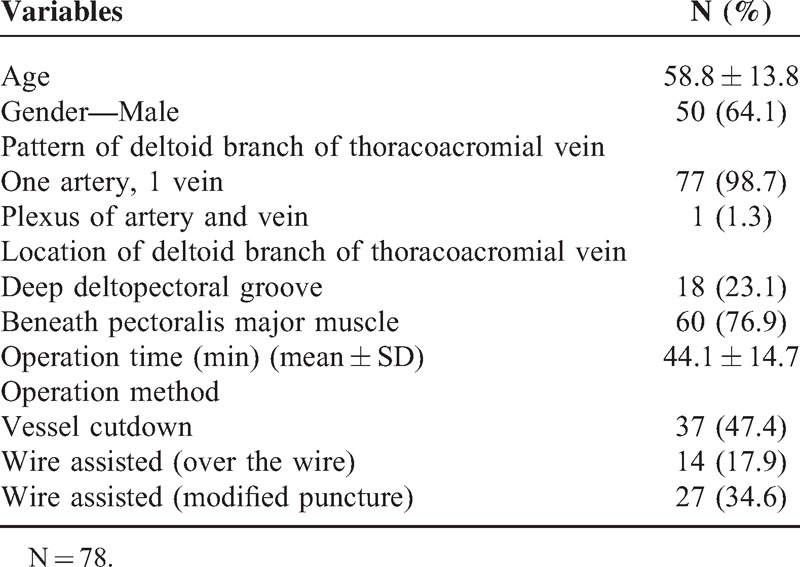
Descriptive Statistics (Entry Vessel = Thoracoacromial vein)

**TABLE 3:**
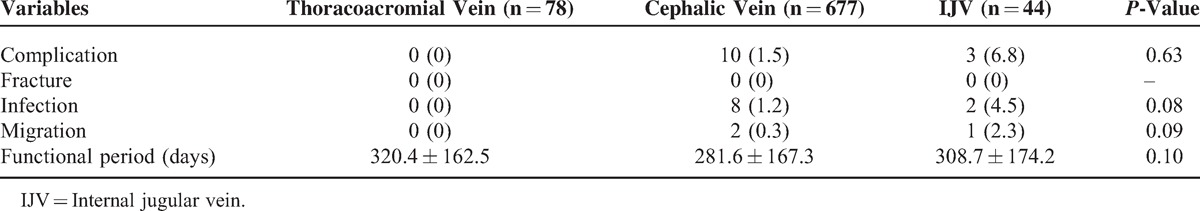
Frequency Between Vessel Entry and Complication, Functional Period

## DISCUSSION

A review of the literature reveals that the thoracoacromial vessel has been widely utilized as the recipient vessel in reconstruction surgery^9–14^ and vascular access for intra-arterial chemotherapy.^15^ There have been no large clinical studies demonstrating the safety and feasibility of intravenous port implantation via the deltoid branch of the thoracoacromial vein. The goal of our study was to identify the location and pattern of the deltoid branch of the thoracoacromial vein and evaluate its feasibility for catheter implantation. Our study identified the location of the deltoid branch of the thoracoacromial vessel as the deltopectoral groove, with the majority (91%) of vessel patterns consisting of 1 artery and 1 vein. Due to variations in anatomy, a suitable deltoid branch of the thoracoacromial vein for catheter implantation could not be identified in 9% of patients. In our study, 9.7% of patients underwent catheter implantation via the deltoid branch of the thoracoacromial vein due to disease or fibrotic cephalic vein, possibly related to aforementioned anatomic variation. From a review of the medical records, the implantation success rate was 100% where a feasible deltoid branch of the thoracoacromial vein was identified. The location of the deltoid branch of the thoracoacromial vein has been further clarified. Only 14.2% of patients had cephalic vein found with the deltoid branches of the thoracoacromial vessel, while the majority (85.8%) of patients were found to have the deltoid branch of the thoracoacromial vessel at the medial aspect of the deltopectoral groove, beneath the pectoralis major muscle. However, a cadaveric study done by Loukas et al demonstrated that 65.2% of the cephalic vein travels with the deltoid branches of the thoracoacromial trunk. This difference may be caused by the dehydration effect of a preservative such as formaldehyde that is used in the preparation of the cadaver. The high percentage of occurrence and relative constant location revealed that the deltoid branch of the thoracoacromial vein could be considered an alternative candidate for catheter implantation.

However, the variable calibers and possible tortuous route of the deltoid branch of the thoracoacromial vein may be a problem if chosen as an entry vessel. In our study, we utilized 3 different implantation methods, according to the caliber of the vessel and its 3-dimensional route. In our study, 37 patients (47/78, 47.4%) with larger calibers and straight vessel routes received the catheter using the vessel cutdown method. In addition, we utilized a metallic wire (Guide Wire M, 0.035 in, 150 cm Terumo Cooperation) to establish catheter implantation in 14 patients (14/78, 17.9%) with tortuous route. After the implantation route was established and confirmed by intraoperative fluoroscopy, the catheter was implanted over the wire. For 27 patients (27/78, 34.6%) with small vessel caliber, we used a smaller metallic wire (V-18 Control Wire, 0.018 in, 200 cm, Boston Scientific) to establish an implantation route and the catheter was implanted with the aid of a subcutaneous dilator with a peel-able sheath. Implantation via the deltoid branch of the thoracoacromial vein could reduce tissue damage when compared with the subclavian and internal jugular vein approach. The subclavian vein puncture requires the creation of a neoroute by the puncture needle and the internal jugular vein puncture requires a subcutaneous tunnel from the neck to the shoulder. In addition, the possibility of iatrogenic pneumothorax and hemothorax cannot be completely avoided during puncture even by an experienced surgeon and under echo-guidance assist.^4,6^ Therefore, the deltoid branch of the thoracoacromial vein can serve as a safe and practical alternative for catheter implantation.

We further compared the functional results and complication rate for different entry vessels. In our experience in 2005 and 2006, 2 iatrogenic pneumothoraces (2/234; 0.85%) occurred, because of the use of subclavian puncture.^3^ Literature review reveals that the risk of inadequate arterial puncture, pneumothorax, and pinch-off symptoms still remains.^16^ In addition, vessel injury caused by subclavian vein puncture could lead to intimal hyperplasia, formation of thrombi, and fibrotic vessel changes. These presentations could subsequently develop into central vein stenosis.^17^ Risk of subclavian puncture complications, including inadequate arterial puncture, pneumothorax, pinch-off symptom, and central vein stenosis, should be minimized. In this study, no iatrogenic pneumothorax or inadequate arterial puncture occurred after principal changes due to our use of the deltoid branch of the thoracoacromial vein as a substitute entry vessel. Furthermore, no catheter fracture occurred in this study, for the following reasons. First, we did not use a port with a metallic fixation device because it would exert larger shear forces on the catheter and lead to fracture.^18^ Second, we created larger pockets for women and obese patients in order to avoid impingement between the locking nut and the catheter.^18^ Third, we avoided subclavian puncture and the risk of pinch-off symptoms, which have been eliminated. In this study, the catheter migration rate was 0.3%, much lower than in our previous study (31/1506, 2%).^19^ This is because we kept the catheter tip 1 cm below the carina under fluoroscopy in order to avoid a shallow catheter tip location.

Some limitations remain in the utilization of the deltoid branch of the thoracoacromial vein. First, size variation and possible tortuous route of the deltoid branch of the thoracoacromial vein is a major concern for catheter implantation. This could be overcome with the assistance of an endovascular device and intraoperative fluoroscopy. In addition, the deltoid branch of the thoracoacromial vein is a good substitute for the subclavian vein with much less operative trauma and lower complication rate. Second, in 9% of patients the deltoid branch of the thoracoacromial vein could not be identified because of anatomical variation. The internal jugular vein approach with echo guidance should be considered as entry route only in patients lacking access to the cephalic vein and deltoid branch of thoracoacromial vein. Because deltoid branch of thoracoacromial vein and cephalic vein were located at neighborhood area and we could explore the two vessels from the same incision. Three different clinical scenarios would be encountered. For patients who were identified these two vessels and those with cephalic vein only, cephalic vein would be the first choice of entry vessel. For those who could be identified deltoid branch of thoracoacromial vein only, deltoid branch of thoracoacromial vein would be utilized for catheter implantation. Only those who absent these two vessels, internal jugular vein was considered as the choice of entry vessel. Therefore, only 5.5% patients needed the internal jugular vein approach for catheter implantation because no feasible cephalic vein or deltoid branch of the thoracoacromial vein was available. Despite these limitations, the deltoid branch of the thoracoacromial vein could provide an alternative entry vessel for patients without a feasible cephalic vein or for those who need repeat port implantation to fit therapeutic needs.

## CONCLUSION

The deltoid branch of the thoracoacromial vein is located in the neighborhood of the cephalic vein. The functional results of intravenous port implantation via the deltoid branch of the thoracoacromial vein are similar to implantation by other entry vessels. It is a safe alternative entry vessel for intravenous port implantation.

## References

[1] Povoski SP A prospective analysis of the cephalic vein cutdown approach for chronic indwelling central venous access in 100 consecutive cancer patients. *Ann Surg Oncol* 2000; 7:496–502. 10947017 10.1007/s10434-000-0496-9

[2] Kincaid EH Davis PW Chang MC Blind” placement of long-term central venous access devices: report of 589 consecutive procedures. *Am Surg* 1999; 65:520–553.discussion 523–524. 10366205

[3] Wu CF Po PJ Wu CY A single-center study of vascular access sites for intravenous ports. *Surg Today* 2014; 44:723–731. 23670039 10.1007/s00595-013-0610-9

[4] Aitken DR Minton JP The pinch-off sign”: a warning of impending problems with permanent subclavian catheters. *Am J Surg* 1984; 148:633–636. 6496853 10.1016/0002-9610(84)90340-4

[5] Hinke DH Zandt-Stastny DA Goodman LR Pinch-off syndrome: a complication of implantable subclavian venous access devices. *Radiology* 1990; 177:353–356. 2217768 10.1148/radiology.177.2.2217768

[6] Biswas S Sidani M Abrol S Emergent median sternotomy for mediastinal hematoma: a rare complication following internal jugular vein catheterization for chemoport insertion—a case report and review of relevant literature. *Case Rep Anesthesiol* 2014; 2014:190172 Epub 2014 Jan 30. 24592335 10.1155/2014/190172 PMC3926366

[7] Plumhans C Mahnken AH Ocklenburg C Jugular versus subclavian totally implantable access ports: catheter position, complications, and interventional pain perception. *Eur J Radiol* 2011; 79:338–342. 20227211 10.1016/j.ejrad.2009.12.010

[8] Loukas M Myers CS Wartmann CT The clinical anatomy of the cephalic vein in the deltopectoral triangle. *Folia Morphol (Warsz)* 2008; 67:72–77. 18335417

[9] Nagaso T Shimizu Y Kasai S Extension of jejunum in the reconstruction of cervical oesophagus with free jejunum transfer using the thoracoacrominal vessel as recipients. *J Plast Recontsr Aesthet Surg* 2012; 65:156–162. 10.1016/j.bjps.2011.08.044 21943681

[10] Al-Mufarrej F Martinez-Jorge J Carlsen BT Use of the deltoid branch-based clavicular head of pectoralis major muscle flap in isolated sternoclavicular infections. *J Plast Recontsr Aesthet Surg* 2013; 66:1702–1711. 10.1016/j.bjps.2013.06.057 23953675

[11] Okada M Ikeda M Uemura T A propeller flap based on the thoracoacrominal artery for reconstruction of a skin defect in the cervical region: a case report. *J Plast Reconstr Aesthet Surg* 2013; 66:720–722. 23021787 10.1016/j.bjps.2012.08.045

[12] Onoda S Sakuraba M Asano T Thoracoacrominal vessels as recipients for head and neck reconstruction and case of vascular complications. *Microsurgery* 2011; 31:628–631. 22025395 10.1002/micr.20947

[13] Walsh MD Bruno AD Onaitis MW Thor role of intrathoracic free flaps for chronic empyema. *Ann Thorac Surg* 2011; 91:865–868. 21353016 10.1016/j.athoracsur.2010.10.019

[14] Eom JS Sun SH Lee TJ Selection of the recipient veins for additional anastomosis of the superficial inferior epigastric vein in breast reconstruction with free transverse rectus abdominis musculocutaneous or deep inferior epigastric artery perforator flaps. *Ann Plast Surg* 2011; 67:505–509. 21407052 10.1097/SAP.0b013e31820bcd5f

[15] Fujii N Yodono H Sasaki T New technique of intra-arterial catheterization via the braches of left axillary artery for continuous infusion chemotherapy. *Nihon Igaku Hoshasen Gakkai Zasshi* 1989; 49:684–687. 2552403

[16] Kim JT Oh TY Chang WH Clinical review and analysis of complications of totally implantable venous access devices for chemotherapy. *Med Oncol* 2012; 29:1361–1364. 21380779 10.1007/s12032-011-9887-y

[17] Agarwal AK Central vein stenosis: current concepts. *Adv Chronic Kidney Dis* 2009; 16:360–370. 19695504 10.1053/j.ackd.2009.06.003

[18] Wu CY Fu JY Feng PH Catheter fracture of intravenous ports and its management. *World J Surg* 2011; 35:2403–2410. 21882033 10.1007/s00268-011-1200-x

[19] Wu CY Fu JY Feng PH Risk factors and possible mechanisms of intravenous port catheter migration. *Eur J Vasc Endovasc Surg* 2012; 44:82–87. 22531452 10.1016/j.ejvs.2012.03.010

